# Full-thickness skin graft versus split-thickness skin graft for radial forearm free flap donor site closure: protocol for a systematic review and meta-analysis

**DOI:** 10.1186/s13643-024-02471-x

**Published:** 2024-02-26

**Authors:** Jasper J.E. Moors, Zhibin Xu, Kunpeng Xie, Ashkan Rashad, Jan Egger, Rainer Röhrig, Frank Hölzle, Behrus Puladi

**Affiliations:** 1 Department of Oral and Maxillofacial Surgery, University Hospital RWTH Aachen, Pauwelsstraße 30, Aachen, 52074 Germany; 2 Institute of Medical Informatics, University Hospital RWTH Aachen, 52074 Aachen, Germany; 3 Cancer Research Center Cologne Essen (CCCE), West German Cancer Center Essen (WTZ), 45122 Essen, Germany; 4 Institute of Artificial Intelligence in Medicine, Essen University Hospital, 45131 Essen, Germany

**Keywords:** Radial forearm free flap, RFFF, Donor site closure, Split-thickness skin graft, Full-thickness skin graft, Donor site morbidity

## Abstract

**Background:**

The radial forearm free flap (RFFF) serves as a workhorse for a variety of reconstructions. Although there are a variety of surgical techniques for donor site closure after RFFF raising, the most common techniques are closure using a split-thickness skin graft (STSG) or a full-thickness skin graft (FTSG). The closure can result in wound complications and function and aesthetic compromise of the forearm and hand. The aim of the planned systematic review and meta-analysis is to compare the wound-related, function-related and aesthetics-related outcome associated with full-thickness skin grafts (FTSG) and split-thickness skin grafts (STSG) in radial forearm free flap (RFFF) donor site closure.

**Methods:**

A systematic review and meta-analysis will be conducted. The Preferred Reporting Items for Systematic Reviews and Meta-Analyses (PRISMA) guidelines will be followed. Electronic databases and platforms (PubMed, Embase, Scopus, Web of Science, Cochrane Central Register of Controlled Trials (CENTRAL), China National Knowledge Infrastructure (CNKI)) and clinical trial registries (ClinicalTrials.gov, the German Clinical Trials Register, the ISRCTN registry, the International Clinical Trials Registry Platform) will be searched using predefined search terms until 15 January 2024. A rerun of the search will be carried out within 12 months before publication of the review. Eligible studies should report on the occurrence of donor site complications after raising an RFFF and closure of the defect. Included closure techniques are techniques that use full-thickness skin grafts and split-thickness skin grafts. Excluded techniques for closure are primary wound closure without the use of skin graft. Outcomes are considered wound-, functional-, and aesthetics-related. Studies that will be included are randomized controlled trials (RCTs) and prospective and retrospective comparative cohort studies. Case-control studies, studies without a control group, animal studies and cadaveric studies will be excluded. Screening will be performed in a blinded fashion by two reviewers per study. A third reviewer resolves discrepancies. The risk of bias in the original studies will be assessed using the ROBINS-I and RoB 2 tools. Data synthesis will be done using Review Manager (RevMan) 5.4.1. If appropriate, a meta-analysis will be conducted. Between-study variability will be assessed using the I^2^ index. If necessary, R will be used. The quality of evidence for outcomes will eventually be assessed using the Grading of Recommendations Assessment, Development and Evaluation (GRADE) approach.

**Discussion:**

This study's findings may help us understand both closure techniques' complication rates and may have important implications for developing future guidelines for RFFF donor site management. If available data is limited and several questions remain unanswered, additional comparative studies will be needed.

**Systematic review registration:**

The protocol was developed in line with the PRISMA-P extension for protocols and was registered with the International Prospective Register of Systematic Reviews (PROSPERO) on 17 September 2023 (registration number CRD42023351903).

**Supplementary Information:**

The online version contains supplementary material available at 10.1186/s13643-024-02471-x.

## Background

### Rationale

Worldwide, head and neck cancer accounts for over 900.000 cases annually [1]. Following ablative surgery, head and neck defects can be reconstructed using microvascular flaps. The radial forearm free flap (RFFF) was first described by Yang et al. in 1981 and is used for various reconstruction purposes [2]. Thanks to its relative thinness, pliability, long and high-calibre pedicle and reliable anatomy, it is one of the workhorses in microvascular reconstruction [3]. However, donor site morbidity such as tendon exposure due to skin graft loss, altered sensitivity and reduced arm function has been reported [4]. These are all factors that could potentially lead to decreased quality of life.

As reconstructive outcomes at the recipient site have improved, a further reduction of morbidity at the donor site has become an essential goal for surgeons [5, 6]. It is believed that the wound closure technique may have an impact on donor site morbidity [4].

When RFFF was first described, Yang et al. suggested donor site closure with a split-thickness skin graft (STSG) [2]. Over the years, various surgical closure techniques have been proposed. Logically, with a small donor site, primary wound closure can be attempted, but this is less common. About 40% of RFFF donor site closure studies report using STSG, and 50% report using full-thickness skin grafts (FTSG), making these the most common closure techniques [4, 7]. Apart from that, other approaches using allogeneic and xenogeneic materials have been described [8–10].

Very recently Mosquera et al. (22/11/2023), Saleki et al (23/11/2023) and Zhang et al. (13/12/2023) published systematic reviews regarding RFFF closure with FTSG vs. STSG [11–13]. However, the conclusions were contradictory. While Mosquera et al. (8 included studies, no meta-analysis) and Saleki et al. (8 included studies, 3-4 in the meta-analysis) concluded improved aesthetics for FTSG with comparable wound-related outcomes, Zhang et al. (13 included studies, 4-6 in the meta-analysis) claimed no benefit in aesthetics with a higher risk of graft failure in FTSG. The reasons for this may be that although all three research groups claim to adhere to the Preferred Reporting Items for Systematic Reviews and Meta-Analyses (PRISMA) statement, there are some important methodological shortcomings and missed opportunities [14].

Mosquera et al. did not register a study protocol, which diminishes transparency, and did not provide a comprehensive search strategy, which potentially led to not identifying all studies meeting the eligibility criteria. Saleki et al. did not provide full search queries for databases, which raises concerns about the replicability, and the number of retrieved records was relatively low (*n =* 78). Zhang et al. conducted statistical syntheses with results from individual studies from different levels of the evidence pyramid and did not provide an explanation or justification for this approach. The authors also used a relatively simple risk of bias assessment, which led to the inclusion of several studies in meta-analyses that either did not provide information on donor site defect size or did not control for differences in donor site defect size between the groups [8, 15–18]. Despite the fact, that RFFF originated in China, where flap surgeons have historically made significant contributions [19], none of the authors included studies in Chinese or searched Chinese databases. Furthermore, none of the authors assessed the quality of evidence using a systematic and transparent framework.

Our systematic review will ensure rigor and comprehensiveness by implementing an exhaustive search strategy, expanding our database coverage, and by including Chinese literature, utilizing advanced risk of bias assessments, and applying the GRADE approach for a robust evaluation of evidence quality. This new systematic review and meta-analysis regarding donor site management after RFFF, with strict adherence to the PRISMA statement would give us reliable evidence for answering the favorable surgical closure technique question, particularly whether STSG or FTSG is preferred. The answer to this question would impact clinical practice and would be very important for developing future clinical guidelines.

### Objective

The objective of our study is to systematically review the literature for evidence on whether a closure technique using FTSG vs STSG is favorable in RFFF donor site management. Outcome parameters will be wound-, function- or aesthetics-related. If the data are sufficient, a meta-analysis will be conducted.

## Methods/design

Our study protocol is developed in line with the Preferred Reporting Items for Systematic Reviews and Meta-Analyses – protocol (PRISMA-P) statement (Additional file 1: Appendix I) [20]. It was registered with the International Prospective Register of Systematic Reviews (PROSPERO) on 17 September 2023 (registration number CRD42023351903). The systematic review and meta-analysis will be reported in line with the PRISMA statement [14].

### Eligibility criteria

The PICO framework and a predefined set of inclusion and exclusion criteria will be used to select studies (Tables 1 and 2):

**Table 1:** PICO statement

P (Patient):	Aged ≥18 years undergoing surgical wound closure of an RFFF donor site
I (Intervention):	Surgical closure using FTSG
C (Comparison):	Surgical closure using STSG
O (Outcome):	Wound-, functional- and aesthetics-related outcomes

**Table 2:** Inclusion and exclusion criteria

Inclusion criteria:	Studies regarding surgical closure technique of the donor site after RFFF
	Studies in English, German and Chinese language
	RCTs, prospective and retrospective comparative cohort studies
	Patients ≥18 years
	Articles from 1981 and younger
	Follow-up ≥3 months
Exclusion criteria:	Cadaveric and animal studies
	Studies regarding OCRFFF

#### Study designs

We will include randomized controlled trials (RCTs), prospective and retrospective comparative cohort studies published in peer-reviewed journals. Case-control studies are neither expected nor included, as the outcome at the level of the hand or wrist will easily be related to the surgery that preceded it. Case series and case reports will be excluded, since they do not have a control group.

#### Participants

We will include studies that examine human adult patients (18 years and older) undergoing surgical wound closure of an RFFF donor site. Animal and cadaveric studies will be excluded. Prophylactic plating has decreased the incidence rate of radial fracture in osteocutaneous radial forearm free flap (OCRFFF) patients [21]. Nevertheless, the procedure is still more invasive than the (fascio)cutaneous RFFF since it uses radial bone to reconstruct a bony defect. Therefore we exclude studies regarding OCRFFF procedures.

#### Intervention

Patients in whom the donor site is surgically closed with FTSG will be in the intervention group.

#### Comparison

The intervention group will be compared to the group in which the donor site is surgically closed with an STSG, as originally suggested by Yang et al. [2].

#### Outcome

Multiple outcome measures will be evaluated. Some outcomes may be reported as individual measures, while other outcomes may be reported as composite measures. We will collect wound outcome measures (haematoma, seroma, partial or complete graft loss on donor site, dehiscence, tendon exposure, delayed healing, need for regrafting, infection), functional outcome measures (pain, sensory deficits, decreased range of motion (ROM), decreased strength, Disability of Arm, Shoulder and Hand (DASH) [22], Mayo wrist score [23], Cold Intolerance Severity Score (CISS) [24]) and aesthetic outcome measures (colouration, thickness, scarring, the Patient and Observer Scar Assessment Scale (POSAS) [25]). Outcomes will be extracted as reported in the included studies (i.e. in dichotomous and continuous data forms).

#### Time frame

In a study regarding scalp reconstruction using FTSG or STSG, the mean healing time was 1.5 weeks for FTSG and 1.9 weeks for STSG [26]. However, the time course of wound healing varies among individuals, and wound healing could therefore take longer [27]. For this reason, studies with a minimum follow-up time of 1 month will be included for wound-related and function-related outcome measures.

Some aesthetically displeasing skin abnormalities appear after a more extended period. Keloid scarring, for example, appears around 3 months after surgery [28]. Therefore, studies with a minimum follow-up time of 3 months will be included for aesthetics-related outcome measures.

#### Setting

There will be no limitations based on the setting type.

#### Language

Articles reported in English, Chinese and German will be included.

### Information sources

A literature search will be performed in multiple databases and search platforms: PubMed, Embase, Scopus, Web of Science, CENTRAL, CNKI). The search will be limited to publications from January 1981, given that the radial forearm flap was first described in 1981. We will rerun the search within 12 months before publication of the review. To maximize the likelihood of finding all relevant literature, we will screen reference lists of included studies identified through the search (backward citation searching), and screen studies that cited the included studies (forward citation searching). These studies will be collected using Citationchaser and a URL reference with a preloaded set of article identifiers will be provided [29].

We will also search several clinical trial registries: ClinicalTrials.gov (clinicaltrials.gov), the German Clinical Trials Register (www.drks.de), the ISRCTN registry (www.isrctn.com) and the International Clinical Trials Registry Platform (trialsearch.who.int).

### Search strategy

The search strategy was developed after consulting two information specialists (I.R. and M.K.; see Acknowledgment). Index terms used in the PubMed search (MeSH) and the Embase search (Emtree) were combined with free-text terms in order to decrease the risk of missing articles that have yet to be indexed or have older indexing. In possibly relevant articles, author keywords were checked and, if applicable, integrated into the search. A text mining tool (PubMed PubReMiner) was used to further improve the draft search. The search query for PubMed is listed in Table 3. Search queries for all databases and search platforms are listed in Additional file 2: Appendix II.

**Table 3:** Search terms for PubMed

**Database**	**Search query**
PubMed	(("Surgical Flaps"[MeSH Terms] OR "Surgical Flap*"[Title/Abstract] OR "flap surgical*"[Title/Abstract] OR "flaps surgical*"[Title/Abstract] OR "radial forearm flap*"[Title/Abstract] OR "radial forearm free flap*"[Title/Abstract]) AND ("Skin Transplantation"[MeSH Terms] OR "Skin Transplantation*"[Title/Abstract] OR "transplantation skin*"[Title/Abstract] OR "grafting skin*"[Title/Abstract] OR "skin graft*"[Title/Abstract] OR "dermatoplast*"[Title/Abstract]) AND ("Forearm"[MeSH Terms] OR "Forearm*"[Title/Abstract] OR "radial*"[Title/Abstract] OR "antebrachi*"[Title/Abstract])) AND (1981:2024/01/15[pdat])

### Study records

#### Data management

Automatic deduplication of records based on Digital Object Identifiers (DOI) will be done using EndNote (Clarivate, Philadelphia, USA). After automatic deduplication, the records will be uploaded to Rayyan (www.rayyan.ai) for further manual deduplication based on identical titles and abstracts by the first and the second reviewer (J.M. and Z.X.) in a blinded manner. Records in Chinese will be handled by the second reviewer (Z.X.) and an additional Chinese-speaking reviewer (K.X.). Records in German will be handled by the first reviewer (J.M.) and an additional German-speaking reviewer (Z.X.). Disagreements are resolved by a third reviewer (B.P.). Then the initial screening of titles and abstracts will take place using Rayyan. The included records will be managed using Citavi 6 (Swiss Academic Software GmbH, Switzerland), software for reference management and knowledge organization.

#### Selection process

Records will be collected by the first reviewer (J.M.). The records identified by the search will be screened for potentially eligible records by title and abstract screening. This process will be independently carried out by the first reviewer (J.M.) and the second reviewer (Z.X.). Records in Chinese will be handled by the second reviewer (Z.X.) and an additional Chinese-speaking reviewer (K.X). Records in German will be handled by the first reviewer (J.M.) and an additional German-speaking reviewer (Z.X.). All data that both reviewers cannot clearly exclude based on its title and abstract receive a full-text review. A study will be included when both reviewers independently assess it as satisfying the inclusion criteria from the full text. If there remains a disagreement after discussion, the third reviewer (B.P.) will mediate. Reasons for excluding trials will be recorded. Both reviewers (J.M. and Z.X or Z.X. and K.X or J.M. and Z.X.) will again assess the included data for the systematic review for inclusion in the meta-analysis. Again, if a disagreement remains after discussion, the third reviewer (B.P.) will mediate. After completion of the selection process, a PRISMA flow diagram will be created.

#### Data collection process

Data will be extracted by the first reviewer (J.M.) and verified by the second reviewer (Z.X.). Data in Chinese will be handled by the second reviewer (Z.X.) and an additional Chinese-speaking reviewer (K.X). Data in German will be handled by the first reviewer (J.M.) and an additional German-speaking reviewer (Z.X.). Disagreements among reviewers will again be settled through discussion, and any that cannot be settled will be decided by the third reviewer (B.P.). Data extraction will be carried out using a data extraction form (Additional file 3: Appendix III). When there are multiple reports of a single study, only the most recent report will be included. We will contact the authors of included studies in case of uncertainties or if important information is absent. Authors will then be contacted through e-mail, with a maximum of three attempts.

### Data items

Variables for which data will be sought are study characteristics (trial design, unit of allocation, start date, end date, trial size, time to follow up, source of financial support), patient characteristics (age, gender, indication for RFFF) and intervention details (donor site defect size in cm^2^, flap type (i.e. cutaneous vs. fasciocutaneous), co-interventions). When possible, we will apply the findings of an intention to treat analysis. If effect sizes cannot be determined, we will contact the authors for additional data.

### Outcomes and prioritization

Outcomes for which data will be sought are listed in Table 4. These outcomes will be wound-related (haematoma, seroma, partial or complete graft loss, dehiscence, tendon exposure, delayed healing, need for re-dressing, infection), function-related (pain, sensory deficits, decreased range of motion (ROM), decreased strength, Disability of Arm, Shoulder and Hand (DASH) [22], Mayo wrist score [23], Cold Intolerance Severity Score (CISS) [24]) and aesthetics-related outcome measures (coloration, thickness, scarring, the Patient and Observer Scar Assessment Scale (POSAS) [25]). Regarding wound-related outcomes minor wound complications are considered haematoma, seroma, partial graft loss, delayed healing, need for re-dressing and major wound complications are considered complete graft loss, dehiscence and tendon exposure.

**Table 4:** Definition of outcomes

**Outcome**	**Measures**
Wound-related	Haemotoma
	Seroma
	Partial or complete graft loss
	Dehiscence
	Tendon exposure
	Delayed healing
	Need for re-dressing
	Infection
Function-related	Pain
	Sensory deficits
	Decreased range of motion (ROM)
	Decreased strength
	Disability of Arm, Shoulder and Hand (DASH) [22]
	Mayo wrist score [23]
	Cold Intolerance Severity Score (CISS) [24]
Aesthetic-related	Coloration
	Thickness
	Scarring
	Patient and Observer Scar Assessment Scale (POSAS) [25]

Primary outcomes will be the wound-related outcomes secondary outcomes will be the aesthetics- and function-related outcomes.

### Risk of bias in individual studies

Risk of bias will be assessed for each included study using the Risk Of Bias In Non-Randomized Studies - of Interventions (ROBINS-I) [30] for non-randomized controlled studies and the Cochrane Risk-of-Bias tool - version 2 (RoB 2) [31] for RCTs. Risk of bias assessment will occur during data extraction. Studies will be independently assessed by the first reviewer (J.M.) and the second reviewer (Z.X.). Studies in Chinese will be assessed by the second reviewer (Z.X.) and an additional Chinese-speaking reviewer (K.X.). Studies in German will be assessed by the first reviewer (J.M.) and an additional German-speaking reviewer (Z.X.). Disagreements will first be settled through discussion, and any that cannot be settled will be decided by the third reviewer (B.P.). If not enough information is provided in the study, the risk of bias will be deemed "unclear", and the study's authors will be contacted for more details.

### Data synthesis

#### Combining different study designs

The study will focus mainly on the systematic review and meta-analysis of RCTs regarding the PICO for every specific outcome measure, as listed in Table 4, rather than a systematic review and meta-analysis of non-randomized controlled trials or a combination of RCTs and non-randomized controlled trials.

**Fig. 1:**
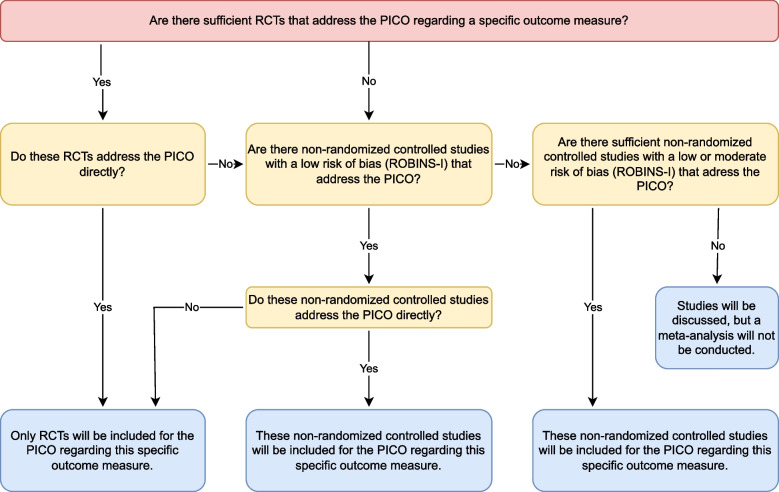
Decision tree for inclusion of non-randomized controlled studies

To decide whether the review should include non-randomized controlled studies, a decision tree adapted from the Cochrane Algorithm will be used [32] (Fig. 1).

When there are sufficient RCTs that address the PICO directly, only RCTs will be included and non-randomized controlled studies will be excluded from the systematic review. When there are insufficient RCTs that address the PICO or only address the PICO indirectly, non-randomized controlled studies with a low overall risk of bias (ROBINS-I) that directly address the PICO will be included. When there are no RCTs that address the PICO or only address the PICO indirectly, non-randomized controlled trials with a low or moderate overall risk of bias (ROBINS-I) will be included. In case there are no RCTs that address the PICO or only address the PICO indirectly, and no non-randomized controlled trials with a low or moderate overall risk of bias (ROBINS-I), then the studies will be discussed, but no meta-analysis will be conducted, since a misleading effect estimate from a systematic review may be more harmful to future patients than no estimate at all, particularly if the people using the evidence to make decisions are unaware of its limitations [32, 33]. In this scenario, the lack of ‘strong’ non-randomized controlled trials could potentially justify a future well-designed RCT.

Studies that meet the eligibility criteria but are not included in the systematic review due to an unacceptable risk of bias will be discussed in the discussion section.

A meta-analysis is only possible if outcomes are reported in at least 2 studies that evaluate the same outcome measures (e.g. tendon exposure), have combinable study designs (Fig. 1) and have comparable interventions (i.e. FTSG), controls (i.e. STSG).

Considering the random effects that are thought to be present in the studies (e.g. the partial subjectivity in the assessment of wounds and aesthetics), a random-effects model will be chosen.

#### Treatment effect

To determine dichotomous data, relative risk (RR) with 95% confidence interval will be used since it is more intuitively interpretable than odds ratio (OR) [34]. Nevertheless, OR has its mathematical advantages. Therefore, OR will be used when a multivariate analysis is conducted. Since morbidity after RFFF is not considered rare, the rare disease assumption cannot be made and OR cannot be regarded as an estimate for RR [4, 34].

To determine continuous data, weighted mean differences (MD) with 95% confidence interval, or standardized mean differences with 95% confidence interval will be used in the case of different measurement scales.

#### Missing data

If desired statistical data like standard deviation or standard error are missing, they will be requested from the author of the original study as described above. If these data cannot be retrieved, they may be reconstructed from other statistical data from the same study.

#### Heterogeneity assessment

To evaluate between-study variability, the I^2^ index will be used (I^2^<25% is usually viewed as low heterogeneity, 25%≤I^2^≤50% as moderate and I^2^>50% as high heterogeneity) [35]. In case of high levels of heterogeneity among the trials (i.e. I^2^>50%), an attempt will be made to explain the heterogeneity by analyzing study characteristics and designs and by subgroup analysis. If applicable, one subgroup analysis will be based on surgical approach for RFFF harvesting (subfascial vs. suprafascial), since suprafascial flap harvesting is believed to have lower risk of donor site complications than subfascial flap harvesting [36]. Another subgroup analysis would be based on mean follow-up period (<1 year vs. ≥1 year), because we expect improvement in function within the first year after surgery, especially as the site heals and rehabilitation progresses. When subgroup analyses are derived from RCTs, the credibility of such analyses in this study will be assessed using the Instrument for assessing the Credibility of Effect Modification Analyses (ICEMAN) [37]. Observational studies, including non-randomized controlled trials, inherently carry a risk of bias. Therefore, to prevent over-optimistic credibility judgments, ICEMAN will not be applied to subgroup analyses derived from non-randomized controlled studies. Assessments will be performed in a blinded fashion by two reviewers (J.M. and Z.X.). Discrepancies that cannot be solved will be mediated by a third reviewer (B.P.).

If quantitative synthesis is not appropriate, a systematic narrative synthesis will be produced. Information will be presented in text and tables to list and describe the characteristics and findings of the included studies. The systematic narrative synthesis will evaluate the findings within the included studies and will also try to evaluate the discrepancies in findings between the included studies.

#### Data synthesis

Data synthesis will be done using Review Manager (RevMan) 5.4.1 (Cochrane Collaboration; www.cochrance.org). R (R Foundation for Statistical Computing; www.R-project.org) will be used if there is a need for more extensive data analysis in conducting the meta-analysis. If we find studies that report multiple outcomes, the Data extraction for complex meta-analysis (DECiMAL) guide will be used, and possibly multivariate analyses will be conducted [38].

### Meta-biases

Reporting bias will be explored using funnel plots if ≥10 studies are included. Testing for funnel plot asymmetry will only be done if standard errors of intervention effect estimates are not similar for all studies. In addition, the test will be interpreted in light of the visual inspection of the funnel plot. Egger's test will be used for funnel plot asymmetry testing in continuous outcomes with intervention effects measured as mean differences [39]. Harbord's test will be used with dichotomous outcomes with intervention effects measured in odds ratios [40]. In the event of dichotomous outcomes with intervention effects measured as relative risk, the funnel plot will only be visually interpreted.

### Confidence in cumulative evidence

The quality of evidence for outcomes will be assessed using the Grading of Recommendations Assessment, Development and Evaluation (GRADE) approach [41]. Assessment will take place in the following domains: risk of bias across studies, inconsistency, imprecision, indirectness and publication bias. The quality of the evidence will then be defined as high (further research is unlikely to change the confidence in estimate of effect), moderate (further research is likely to have an important impact on confidence in estimate of effect and may change the estimate), low (further research is very likely to have an important impact on confidence in estimate of effect & is very likely to change the estimate) or very low (any estimate of effect is very uncertain). A summary of findings (SoF) table will be created with a maximum of 7 outcomes (major wound complications, minor wound complications, complete skin graft loss, tendon exposure, functional outcome, patient-reported aesthetic outcome and observer-reported aesthetic outcome) using the GRADEpro GDT software [42]. Possible narrative reviews of described outcomes will also be added to the SoF table. Guideline development is beyond the scope of this systematic review.

### Supplementary Information

**Additional file 1. **PRISMA-P 2015 Checklist.

**Additional file 2. **Search strategy.

**Additional file 3. **Data extraction form.

## Data Availability

Not applicable.

## Abbreviations

CNKI: China National Knowledge Infrastructure
CENTRAL: Cochrane Central Register of Controlled Trials
CISS: Cold Intolerance Severity Score
DECiMAL: Data Extraction for Complex Meta-Analysis
DOI: Digital Object Identifier
DASH: Disability of Arm, Shoulder and Hand
FTSG: Full-Thickness Skin Graft
GRADE: Grading of Recommendations Assessment, Development and Evaluation
ICEMAN: Instrument for assessing the Credibility of Effect Modification Analyses
MD: Mean Difference
OCRFFF: Osteocutaneous Radial Forearm Free Flap
OR: Odds Ratio
PROSPERO: International Prospective Register of Systematic Reviews
POSAS: Patient and Observer Scar Assessment Scale
PICO: Patient, Intervention, Comparison, Outcome
PRISMA: Preferred Reporting Items for Systematic Reviews and Meta-Analyses
RCT: Randomized Controlled Trial
RFFF: Radial Forearm Free Flap
ROM: Range Of Motion
RoB 2: Cochrane Risk-of-Bias tool version 2
ROBINS-I: Risk Of Bias In Non-Randomized Studies of Interventions
RR: Relative Risk
SoF: Summary of Findings
STSG: Split-Thickness Skin Graft
