# Supplementary material for: Full-thickness skin graft versus split-thickness skin graft for radial forearm free flap donor site closure: protocol for a systematic review and meta-analysis
Source: Syst Rev. 2024 Feb 26;13:74. doi: 10.1186/s13643-024-02471-x (PMC10895847; doi:10.1186/s13643-024-02471-x)
Supplement: Supplementary file 2 — Additional file 2. Search strategy. [file 13643_2024_2471_MOESM2_ESM.docx]

Appendix II: Search strategy

| **Database** | **Search query** |
| --- | --- |
| PubMed | (("Surgical Flaps"[MeSH Terms] OR "Surgical Flap*"[Title/Abstract] OR "flap surgical*"[Title/Abstract] OR "flaps surgical*"[Title/Abstract] OR "radial forearm flap*"[Title/Abstract] OR "radial forearm free flap*"[Title/Abstract]) AND ("Skin Transplantation"[MeSH Terms] OR "Skin Transplantation*"[Title/Abstract] OR "transplantation skin*"[Title/Abstract] OR "grafting skin*"[Title/Abstract] OR "skin graft*"[Title/Abstract] OR "dermatoplast*"[Title/Abstract]) AND ("Forearm"[MeSH Terms] OR "Forearm*"[Title/Abstract] OR "radial*"[Title/Abstract] OR "antebrachi*"[Title/Abstract])) AND (1981:2024/01/15[pdat]) |
| Embase | ('surgical flaps'/exp OR 'surgical flap*':ti,ab,kw OR 'flaps surgical*':ti,ab,kw OR 'radial forearm flap'/exp OR 'radial forearm flap*':ti,ab,kw OR 'radial forearm free flap*':ti,ab,kw) AND ('skin transplantation'/exp OR 'skin transplant*':ti,ab,kw OR 'transplantation skin*':ti,ab,kw OR 'grafting skin':ti,ab,kw OR 'skin graft*':ti,ab,kw OR 'dermatoplast*':ti,ab,kw) AND ('forearm'/exp OR 'forearm*':ti,ab,kw OR 'radial*':ti,ab,kw OR 'antebrachi*':ti,ab,kw) AND [1981-2024]/py |
| Scopus | TITLE-ABS-KEY(("surgical flaps" OR "flaps surgical" OR "radial forearm flap" OR "radial forearm free flap") AND ("Skin Transplantation" OR "transplantation skin" OR "graft* skin" OR "skin graft*" OR "dermatoplast*") AND ("forearm" OR "radial*" OR "antebrachi*")) AND PUBYEAR > 1980 |
| Web of Science | TS=("surgical flap*" OR "flaps surgical*" OR "flap surgical*" OR "radial forearm flap*" OR "radial forearm free flap*") AND TS=("skin transplant*" OR "transplantation skin*" OR "transplantations skin*" OR "grafting skin*" OR "skin graft*" OR "dermatoplast*") AND TS=("forearm*" OR "radial*" OR "antebrachi*") AND DOP=1981-01-01/2024-01-15 |
| CENTRAL | #1 MeSH descriptor: [Surgical Flaps] explode all trees  #2 (Surgical Flap*):ti,ab,kw  #3 MeSH descriptor: [Skin Transplantation] explode all trees  #4 ((Skin Transplant*) OR (Skin Graft*) OR Dermatoplast* OR (Skin plast*)):ti,ab,kw  #5 MeSH descriptor: [Forearm] explode all trees  #6 Forearm* OR Radial* OR Antebrach*  **#7 (#1 OR #2) AND (#3 OR #4) AND (#5 OR #6)** |
| CNKI | (FT%="前臂” AND FT%="桡侧") OR SU%="前臂桡侧游离皮瓣" OR SU%="前臂桡侧皮瓣"  *[English: (FT%="forearm" AND FT%="radial") OR SU%="forearm radial free flap" OR SU%="forearm radial flap"]* |
