# Supplementary material for: Full-thickness skin graft versus split-thickness skin graft for radial forearm free flap donor site closure: protocol for a systematic review and meta-analysis
Source: Syst Rev. 2024 Feb 26;13:74. doi: 10.1186/s13643-024-02471-x (PMC10895847; doi:10.1186/s13643-024-02471-x)
Supplement: Supplementary file 3 — Additional file 3. Data extraction form. [file 13643_2024_2471_MOESM3_ESM.docx]

Data extraction form for intervention reviews: RCTs and non-randomized clinical studies

Instructions on using this data extraction form^[[1]](#footnote-1)^:

- This data extraction form is used in studies that receive a full-text screening, i.e., that were not excluded after title & abstract screening.
- Any missing information must be reported as unclear or not described, so it is clear that extraction of this part of the data is not forgotten.
- Do not proceed with data extraction if the study is excluded based on eligibility criteria.

| Review title or ID |  |
| --- | --- |
| Study ID *(surname of first author and year first full report of study was published e.g. Smith 2001)* |  |
| Report ID |  |
| Report ID of other reports of this study including errata or retractions |  |
| Notes | |

# General Information

| Date form completed *(dd/mm/yyyy)* |  |
| --- | --- |
| Name/ID of person extracting data |  |
| Reference citation |  |
| Study author contact details |  |
| Publication type *(e.g. full report, abstract, letter)* |  |
| Notes: | |

# Study eligibility

| Study Characteristics | Eligibility criteria | | Eligibility criteria met? | | | Location in text or source *(pg & ¶/fig/table/other)* |
| --- | --- | --- | --- | --- | --- | --- |
|  |  | | Yes | No | Unclear |  |
| Type of study | Randomised Controlled Trial | |  |  |  |  |
|  | Quasi-randomised Controlled Trial | |  |  |  |  |
|  | Prospective comparative cohort study | |  |  |  |  |
|  | Retrospective comparative cohort study | |  |  |  |  |
|  | Other design (specify): | |  |  |  |  |
| Participants |  | |  |  |  |  |
| Types of intervention |  | |  |  |  |  |
| Types of comparison |  | |  |  |  |  |
| Types of outcome measures |  | |  |  |  |  |
| INCLUDE | | EXCLUDE | | | | |
| Reason for exclusion |  | | | | | |
| Notes: | | | | | | |

**DO NOT PROCEED IF STUDY IS EXCLUDED FROM REVIEW**

# Characteristics of included studies

## Methods

|  | **Descriptions as stated in report/paper** | **Location in text or source** *(pg & ¶/fig/table/other)* |
| --- | --- | --- |
| **Study design** *(RCT, prospective comparative cohort study or retrospective comparative cohort study)* |  |  |
| **Unit of allocation** *(by individuals, cluster/ groups or body parts)* |  |  |
| **Start date** |  |  |
| **End date** |  |  |
| **Trial size** |  |  |
| **Time to follow up** |  |  |
| **Source of financial support** |  |  |
| **Notes:** | | |

## Participants

|  | Description  *Include comparative information for each intervention or comparison group if available* | Location in text or source *(pg & ¶/fig/table/other)* |
| --- | --- | --- |
| Age |  |  |
| Gender |  |  |
| Indication for radial forearm free flap |  |  |
| Notes: | | |

## Intervention groups

*Copy and paste table for each intervention and comparison group*

**Intervention Group 1**

|  | Description as stated in report/paper | Location in text or source *(pg & ¶/fig/table/other)* |
| --- | --- | --- |
| Group name |  |  |
| Number of participants randomised to group |  |  |
| Donor site defect size in cm^2^ |  |  |
| Flap type *(i.e. cutaneous or fasciocutaneous)* |  |  |
| Co-interventions |  |  |
| Notes: | | |

## Outcomes

*Copy and paste table for each outcome.*

**Outcome 1**

|  | Description as stated in report/paper | | | Location in text or source *(pg & ¶/fig/table/other)* |
| --- | --- | --- | --- | --- |
| Outcome | Wound-related | Function-related | Aesthetics-related |  |
| Outcome definition *(as listed in Table 4 study protocol)* |  | | |  |
| Device used for measuring *(if relevant)* |  | | |  |
| Unit of measurement *(if relevant)* |  | | |  |
| Notes: | | | | |

# Data and analysis

*Copy and paste the appropriate table for each outcome. Delete table if not applicable.*

### For RCT and non-randomized controlled study: dichotomous outcome

|  | Description as stated in report/paper | | | | Location in text or source *(pg & ¶/fig/table/other)* |
| --- | --- | --- | --- | --- | --- |
| Comparison |  | | | |  |
| Outcome |  | | | |  |
| Results | Intervention | | Comparison | |  |
|  | No. with event | Total in group | No. with event | Total in group |  |
|  |  |  |  |  |  |
| Any other results reported *(e.g. odds ratio, risk difference, CI or P value)* |  | | | |  |
| No. missing participants |  | |  | |  |
| Reasons missing |  | |  | |  |
| No. participants moved from other group |  | |  | |  |
| Reasons moved |  | |  | |  |
| Unit of analysis *(by individuals, cluster/groups or body parts)* |  | | | |  |
| Statistical methods used and appropriateness of these *(e.g. adjustment for correlation)* |  | | | |  |
| Notes: | | | | | |

### For RCT and non-randomized controlled study: continuous outcome

|  | | Description as stated in report/paper | | | | | Location in text or source *(pg & ¶/fig/table/other)* |
| --- | --- | --- | --- | --- | --- | --- | --- |
| Comparison | |  | | | | |  |
| Outcome | |  | | | | |  |
| Results | Intervention | | | Comparison | | |  |
|  | Mean | SD *(or other variance, specify)* | No. participants | Mean | SD *(or other variance, specify)* | No. participants |  |
|  |  |  |  |  |  |  |  |
| Any other results reported *(e.g. mean difference, CI, P value)* | |  | | | | |  |
| No. missing participants | |  | |  | | |  |
| Reasons missing | |  | |  | | |  |
| No. participants moved from other group | |  | |  | | |  |
| Reasons moved | |  | |  | | |  |
| Unit of analysis *(individuals, cluster/ groups or body parts)* | |  | | | | |  |
| Statistical methods used and appropriateness of these *(e.g. adjustment for correlation)* | |  | | | | |  |
| Notes: | | | | | | | |

### For RCT and non-randomized controlled study: other outcome

|  | Description as stated in report/paper | | | | Location in text or source *(pg & ¶/fig/table/other)* |
| --- | --- | --- | --- | --- | --- |
| Comparison |  | | | |  |
| Outcome |  | | | |  |
| No. participant | Intervention | | Control | |  |
|  |  | |  | |  |
| Results | Intervention result | SE (or other variance) | Control result | SE (or other variance) |  |
|  |  |  |  |  |  |
|  | Overall results | | SE (or other variance) | |  |
|  |  | |  | |  |
| Any other results reported |  | | | |  |
| No. missing participants |  | |  | |  |
| Reasons missing |  | |  | |  |
| No. participants moved from other group |  | |  | |  |
| Reasons moved |  | |  | |  |
| Unit of analysis *(by individuals, cluster/groups or body parts)* |  | | | |  |
| Statistical methods used and appropriateness of these |  | | | |  |
| Notes: | | | | | |

# Other information

|  | **Description as stated in report/paper** | **Location in text or source** *(pg & ¶/fig/table/other)* |
| --- | --- | --- |
| **Key conclusions of study authors** |  |  |
| **References to other relevant studies** |  |  |
| **Correspondence required for further study information** *(from whom, what and when)* |  | |
| **Notes:** | | |

# Definitions^[[2]](#footnote-2),^ ^[[3]](#footnote-3)^

| Bias | A systematic error or deviation in results or inferences from the truth. In studies of the effects of health care, the main types of bias arise from systematic differences in the groups that are compared (selection bias), the care that is provided, exposure to other factors apart from the intervention of interest (performance bias), withdrawals or exclusions of people entered into a study (attrition bias) or how outcomes are assessed (detection bias). Reviews of studies may also be particularly affected by reporting bias, where a biased subset of all the relevant data is available. |
| --- | --- |
| Clusters | A group of participants who have been allocated to the same intervention arm together, as in a cluster-randomised trial, e.g. a whole family, town, school or patients in a clinic may be allocated to the same intervention rather than separately allocating each individual to different arms. |
| Exclusions | Participants who were excluded from the study or the analysis by the investigators. |
| Quasi-randomised controlled trial | A study in which the method of allocating people to intervention arms was not random, but was intended to produce similar groups when used to allocate participants. Quasi-random methods include: allocation by the person's date of birth, by the day of the week or month of the year, by a person's medical record number, or just allocating every alternate person. |
| Unit of allocation | The unit allocated to an intervention arm. In most studies individual participants will be allocated, but in others it may be individual body parts (e.g. different teeth or joints may be allocated separately) or clusters of multiple people. |
| Unit of analysis | The unit used to calculate N in an analysis, and for which the result is reported. This may be the number of individual people, or the number of body parts or clusters of people in the study. |
| Unit of measurement | The unit in which an outcome is measured, e.g. height may be measured in cm or inches; depression may be measured using points on a particular scale. |
| Withdrawals | Participants who voluntarily withdrew from participation in a study before the completion of outcome measurement. |

1. Adapted from the Cochrane Developmental, Psychosocial and Learning Problems (DPLP) Review Group data extraction form template, Version 3, April 2014. Available at: https://dplp.cochrane.org/data-extraction-forms (Accessed: 17 October 2022) [↑](#footnote-ref-1)
2. Cochrane Collaboration Glossary, 2010. Available at: www.cochrane.org/glossary [↑](#footnote-ref-2)
3. Last JM (editor), A Dictionary of Epidemiology, 4th Ed. New York: Oxford University Press, 2001. [↑](#footnote-ref-3)
